# Aminaphtone therapy in patients with type 1 diabetes and albuminuria: a case report

**DOI:** 10.1186/1752-1947-8-443

**Published:** 2014-12-19

**Authors:** Concetta Romano, Consuelo Tamburella, Martino Costa, Marco Messina, Anna Lisa Fassari, Marco Bertini

**Affiliations:** 18 Department of Cardiology, Acireale Hospital, Provincial Health Centre of Catania, Catania, Italy; 19 Department of Ophthalmology, Acireale Hospital, Provincial Health Centre of Catania, Catania, Italy; 20 Medical Department, Laboratori Baldacci SpA, Via San Michele degli Scalzi 73, 56124 Pisa, Italy

**Keywords:** Insulin-dependent diabetes mellitus, Microangiopathy

## Abstract

**Introduction:**

Microalbuminuria in type 1 diabetes is the earliest manifestation of diabetic microangiopathy (nephropathy). To date, the pharmacological approach to microangiopathy has not been shown to be useful. By using aminaphtone to control nephrologic complications of insulin-dependent diabetes mellitus we first obtained a significant improvement in microalbuminuria confirming this new pharmacological approach for insulin-dependent diabetes mellitus organospecific complications control.

**Case presentation:**

After being treated with standard therapy for insulin-dependent diabetes mellitus (insulin) for more than 20 years, a 49-year-old white man affected by insulin-dependent diabetes mellitus adopted the standard therapy aminaphtone for a period of 2 months.

This therapy allowed a significant reduction of proteinuria from baseline evaluation that immediately increased after he stopped aminaphtone therapy.

**Conclusions:**

Aminaphtone therapy, used globally in the treatment and prevention of endothelial dysfunctions, could be an interesting option for patients with insulin-dependent diabetes mellitus with the express purpose of preventing diabetic nephropathy.

## Introduction

Microangiopathic complications of diabetes include retinopathy, nephropathy and neuropathy. These complications are specific to diabetes and arise as a result of hyperglycaemia that lasts a long time. Undoubtedly, other metabolic factors, and environmental and genetic factors are involved in the pathogenesis. Diabetic nephropathy affects 20 to 40% of patients with insulin-dependent diabetes mellitus (IDDM), particularly those with onset before puberty [1] and, probably, those with a hereditary predisposition to hypertension [2]. Patients with diabetes with incipient nephropathy (persistent microalbuminuria) have a 5 to 10 times greater risk of developing proliferative retinopathy than those without albuminuria; furthermore, diabetic nephropathy is almost invariably accompanied by retinopathy. The functional alterations that occur early in the natural history of diabetic nephropathy include microalbuminuria and glomerular hyperfiltration. Microalbuminuria is defined as a subclinical increase in the rate of urinary albumin excretion in the range of 30 to 300mg/day. Microalbuminuria is due to the increased permeability of the glomerular capillaries, probably secondary to increased glomerular capillary pressure [3] and to the loss of negative charge at the level of the glomerular basement membrane.

Patients with IDDM with microalbuminuria have a 20 times greater risk of developing a clinical nephropathy compared to those with a normal albumin excretion [4]. Microalbuminuria is considered a risk factor for diabetic nephropathy and progressive renal failure in diabetes [5–8]. Longitudinal and cross-sectional studies conducted on type 2 diabetes have allowed the identification of risk factors associated with the development of microalbuminuria and the progression of microalbuminuria to diabetic nephropathy. These include: low body mass index, early onset of diabetes, hyperglycaemia, hypertension, dyslipidaemia, cigarette smoking, and a family history of hypertension [9–14]. Although microalbuminuria is considered by many authors to be the early stage of an irreversible process, recent research has not confirmed this view, showing that microalbuminuria often returns to its normal value [15, 16]. The histopathological features characteristic of diabetic kidney disease occur in the glomerulus. The main changes are: increase in the thickening of the glomerular basement membrane, increase of volume of the mesangium, the presence of hyaline deposits and global glomerular sclerosis [17]. The glomerular filtration rate (GFR) is closely related to the surface of the basal membrane of the glomerular capillaries (filtration surface) which is in turn determined by the number of clusters present at the time of diagnosis, the entity of the expansion of the mesangium, the expansion capacity and the number of sclerotic glomeruli. Urinary albumin excretion is related to the size of the pores of filtration. The vascular endothelium plays a central role in the regulation of vascular tone. Endothelin (ET) is a potent vasoconstrictor produced by endothelium that contributes to basal vascular tone. Vasoconstriction in response to altered endogenous ET may lead to hyperperfusion and subsequent microvascular damage. Hyperperfusion is one of the keys to the onset and progression of microvascular complications in diabetes. Such a haemodynamic condition can be determined by the excessive release of vasodilator substances, as for example during ketonic decompensation, or for a reduced action of vasoconstrictive substances. This interpretation may be the pathophysiological basis of what has been observed in diabetes. In patients with diabetes there is a deficit of action of ET that, unlike in the normal patient, does not induce vasoconstriction. In fact, individuals with diabetes have levels of ET that would appear to be determined mainly by triglycerides and insulin.

Aminaphtone (2-hydroxy-3-methyl-1,4-napthohydroquinone-2-p-aminobenzoate) is a synthetic molecule derived from four aminobenzoic acids which is currently employed for “capillary disorders” and for chronic venous insufficiency [18]. This drug has recently demonstrated the ability to downregulate ET-1 production in ECV304 cells by interfering with transcription of preproET-1 (PPET-1) gene expression [19]. At the same time, cytofluorometry has shown that aminaphtone significantly reduces the expression of E-selectin (endothelial-leukocyte adhesion molecule 1; ELAM-1) both in resting and in ET-B -activated ECV304 cells in a dose-dependent manner [20]. *In vivo*, in patients affected by systemic sclerosis, 12 weeks of aminaphtone treatment has demonstrated the ability to downregulate sELAM-1 (soluble E-selectin adhesion molecules 1) and sVCAM-1 (soluble vascular cell adhesion molecule 1) [21]. In a rat model of monocrotaline-induced pulmonary hypertension, the administration of aminaphtone (30mg/kg/day or 150mg/kg/day) significantly lowered rat mortality and significantly reduced plasma ET-1 concentration [22]. Aminaphtone has also demonstrated antiphlogistic activity on endothelial cells [23]. In order to control vascular microangiopathy in patients affected by IDDM with proteinuria and hypertension, we added aminaphtone to standard therapy for IDDM (insulin) and hypertension (angiotensin-converting enzyme inhibitors).

## Case presentation

**Figure 1:**
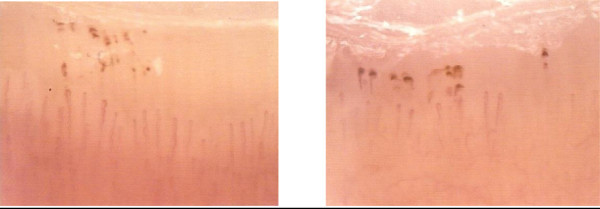
**Periungual videocapillaroscopic: microbleeds spread “pearl parades**”**.**

**Figure 2:**
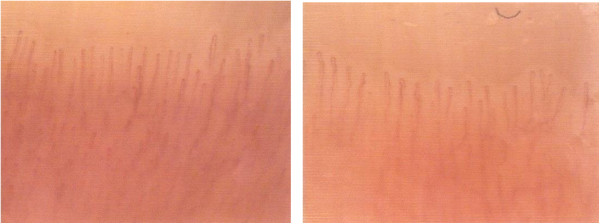
**Periungual videocapillaroscopic: resolution of profuse bleeding.**

A 49-year-old white man with type 1 diabetes has, from the age of 20, been treated with NovoRapid^®^ (insulin aspart; 3U breakfast time, 8U lunch time, and 10U dinner time) and Lantus^®^ (insulin glargine; 14U 10 p.m.) for glycaemic control for more than 20 years. He has been hypertensive since 2006 and receives treatment with ramipril: one tablet 5mg at breakfast time. He has also been treated with Torvast (atorvastatin; one tablet 10mg/day) for hypercholesterolaemia. Microalbuminuria has been present in this patient since 2006 independently of any pharmacological treatments; no alterations have been reported in his fundus oculi during the time. An examination of his nailfold periungual videocapillaroscopy shows the presence of beanpole loops with dilated venous tract and presence of pearl-like microbleeds with clear signs of microangiopathy (Figure 1). His microalbuminuria value was 487.1mg/L; GFR was under 58mL/minute; creatinine was 1.31mg/dL and arterial blood pressure was 140/85mmHg. He began treatment with aminaphtone 75mg one tablet twice a day for 2 months. After 2 months of therapy he was re-evaluated by videocapillaroscopic examination showing a clear resolution of profuse bleeding (Figure 2). At the same time his microalbuminuria value significantly decreased (98.3mg/L); his GFR (under 57mL/minute) and creatinine (1.33mg/dL) were substantially unchanged but his arterial blood pressure decreased to 130/80mmHg. Taking into account the results obtained with the above treatment, he continues to take aminaphtone; however, intake was spontaneously interrupted for 2 months. After 2 months without aminaphtone treatment he repeated proteinuria evaluation: his microalbuminuria significantly increased to 573.0mg/L while his GFR (under 58mL/minute) and creatinine (1.31mg/dL) were unchanged and his arterial blood pressure again increased to 140/85mmHg. No side effects related to aminaphtone were detected during the trial.

## Discussion

This case report initially describes a significant reduction of proteinuria in a patient with diabetes type 1, due to the use of aminaphtone. Our patient was first evaluated after 2 months of treatment (aminaphtone capsules 75mg twice a day), showing a significant decrease in microalbuminuria (from 487.1mg/dL to 98.3mg/dL = −388.8mg/dL) confirmed by an improvement in the capillaroscopic examination, while GFR and creatinine remained substantially unchanged. Surprisingly, arterial blood pressure decreased from 140/85mmHg to 130/80mmHg after treatment. Subsequently, he continued treatment with aminaphtone for 2 months, but spontaneously interrupted the treatment. He was re-evaluated 2 months after discontinuation of the drug and, again, showed an increase in proteinuria (from 98.3mg/dL to 573mg/dL = +474.7mg/dL), substantially the same GFR and creatinine values, and a new increase in arterial blood pressure to 140/85mmHg: this kind of re-challenge clearly demonstrates the relationship between proteinuria and aminaphtone therapy. The recent preclinical findings about aminaphtone (anti-ET-1 and anti-E-selectin activities together with a significant endothelial antiphlogistic effect) [19–23] seem to correlate with this result, confirming that microangiopathy is the leading cause of microalbuminuria in patients with IDDM.

## Conclusions

Aminaphtone therapy seems to be useful for proteinuria control in patients with type 1 diabetes. The correlation between proteinuria and arterial blood pressure that significantly decreased after aminaphtone treatment and increased once treatment was stopped was surprising. To the best of our knowledge, descriptions of aminaphtone’s activity have not included a report of a novel therapeutic option. For the purposes of therapeutic effectiveness, the duration of treatment (more than 2 months) together with the compliance of daily administration of the drug appears to be relevant. Further controlled studies, including randomised double-blind controlled trials, are needed to definitively evaluate aminaphtone’s efficacy over the long term in patients with IDDM. Nevertheless, the lesson to take from this case report is that a pharmacological treatment of diabetic microangiopathy with a vascular drug like aminaphtone could be useful for proteinuria control postponing IDDM nephrologic complications.

## Consent

Written informed consent was obtained from the patient for publication of this case report and accompanying images. A copy of the written consent is available for review by the Editor-in-Chief of this journal.

## Abbreviations

ET: Endothelin
GFR: Glomerular filtration rate
IDDM: Insulin-dependent diabetes mellitus.
